# Synthesis of Amphiphilic Statistical Copolymers Bearing Methoxyethyl and Phosphorylcholine Groups and Their Self-Association Behavior in Water

**DOI:** 10.3390/polym12081808

**Published:** 2020-08-12

**Authors:** Thi Lien Nguyen, Yuuki Kawata, Kazuhiko Ishihara, Shin-ichi Yusa

**Affiliations:** 1 Department of Applied Chemistry, Graduate School of Engineering, University of Hyogo, 2167 Shosha, Himeji, Hyogo 671-2280, Japan; nguyenlienk56hh@gmail.com (T.L.N.); Yuuki.Kawata@sekisui-fuller.com (Y.K.); 2 Department of Materials Engineering, School of Engineering, The University of Tokyo, 7-3-1 Hongo, Bunkyo-ku, Tokyo 113-8656, Japan; ishihara@mpc.t.u-tokyo.ac.jp

**Keywords:** statistical copolymer, 2-methoxyethyl acrylate, 2-methacryloyloxyethyl phosphorylcholine, self-association behavior

## Abstract

Biocompatible amphiphilic statistical copolymers P(MEA/MPC*_m_*) composed of 2-methoxyethyl acrylate (MEA) and 2-methacryloyloxyethyl phosphorylcholine (MPC) were prepared with three different mol% of the hydrophilic unit MPC (*m* = 6, 12 and 46 mol%). The monomer reactivity ratios of MEA (*r*_MEA_) and MPC (*r*_MPC_) were 0.53 and 2.21, respectively. The *r*_MEA_ × *r*_MPC_ value of 1.17 demonstrated that statistical copolymerization was successful. P(MEA/MPC_12_) and P(MEA/MPC_46_) copolymers did not undergo aggregation in water, whereas the P(MEA/MPC_6_) copolymer formed micelles in water with a hydrodynamic radius (*R*_h_) of 96.9 nm and a critical aggregation concentration, which was determined using pyrene fluorescence, at 0.0082 g/L. The restricted motion of the protons in the hydrophobic MEA units in the micelles’ cores provided additional evidence of self-association in P(MEA/MPC_6_).

## 1. Introduction

The self-association of random copolymers has become increasingly important in drug delivery systems [1,2,3], imaging techniques [4,5,6,7] and many other fields [8]. The self-association behavior of amphiphilic random copolymers is more complicated than that of block polymers because of their ill-defined, randomly arranged monomer sequence. As such, the association between the hydrophobic units of a single polymer chain can form unimer micelles, and is independent of the polymer’s concentration or the presence of interpolymer micelles, which are generated via the interaction of multiple polymer chains above the critical aggregation concentration (CAC). Morishima et al. [9] investigated the self-organization of random copolymers of sodium 2-(acrylamido)-2-methylpropanesylfonate (AMPS) and methacrylamides bearing bulky hydrophobic groups, including *n*-dodecyl (LA), cyclododecyl (CD) and 1-adamantyl (AD) groups. Here, the aggregates formed from these random copolymers in aqueous solutions were due to intra and/or intermolecular self-association. The copolymers of AMPS and CD or AD tended to form unimer micelles up to a concentration limit of ca. 7 wt.%; conversely, the LA-containing copolymers self-associated to form unimers at concentrations below 0.2 wt.% and interpolymer aggregates at higher concentrations. These results showed that the chemical structure of the hydrophobic monomer in random copolymers was a critical factor in the self-association process. Additionally, the self-association of random copolymers depends on other factors, such as the type of solvent, the hydrophobic content in the copolymer [10,11], the distribution of the hydrophobic groups along the polymer chains [12], and the prevailing protocol for micellization [13]. Recently, Terashima et al. [11] reported on amphiphilic random methacrylate copolymers composed of poly(ethylene glycol) methacrylate and alkyl methacrylate (RMA) with linear alkyl chains that were 1–18 carbon units long. Here, copolymers with an RMA content of 20–40% tended to undergo single-chain folding even at polymer concentrations of up to ~6 wt.%. Generally, the copolymers tended to exist as unimer micelles in water when the concentration of dodecyl methacrylate (DMA) was less than 40 mol%, and as multi-chain aggregates when the concentration of DMA was over 50 mol%. Neal et al. [14] showed that the copolymer’s composition was important to the mean nano-object size of the structures formed from random copolymers. Here, a series of poly(*n*-butyl methacrylate-*stat*-methacrylic acid) (P(BMA-*stat*-MAA) statistical copolymers with various BMA/MAA compositions (i.e., from 77:23 to 93:7) was synthesized. It was found that the radii of the nano-objects formed by the self-assembly of the copolymers were independent of the copolymer’s molecular weight, but inversely proportional to the hydrophilic methyl methacrylate (MMA) content. In other words, fewer MAA units in the copolymer chain resulted in the formation of larger nano-objects. Since the polymer’s micelles were formed with a hydrophobic core and a hydrophilic shell, molecules that exhibited poor water solubility, such as pharmaceutical drugs, could be encapsulated, transported, and protected from the surrounding environment. Moreover, this tactic minimized the associated side effects of the drug and extended its circulation time [15].

The formation of micelles in water was investigated for applications in biological environments. Here, the hydrophobic domain of the polymer’s micelles encapsulated the hydrophobic drugs and imaging moieties, while the outer hydrophilic shell exhibited biocompatibility with various biological applications. Hydrophobic poly(2-methoxyethyl acrylate) (PMEA) showed excellent biological and blood compatibility, and inhibited the adsorption of proteins [16,17,18,19]. Hoshiba et al. [16] reported that the surface of PMEA underwent weak interactions with proteins and blood components due to the presence of “intermediate water” molecules in the polymer that crystallized at elevated temperatures. However, very little is known about the mechanism of expression of hemocompatibility in PMEA. Tanaka et al. [18] reported that the MEA content in MEA/2-hydroxyethyl methacrylate copolymers was an important parameter affecting the state of the water molecules in the polymer, ultimately influencing platelet compatibility. The lowest values associated with the adhesion number and the morphological changes in the platelets on the copolymer’s surface were observed in copolymers containing 80–100 mol% MEA. Conversely, the copolymers with less than 60 mol% MEA exhibited poor biocompatibility. Furthermore, PMEA has a glass transition temperature as low as −50 °C, is soluble in organic solvents, and exhibits traits such as hydrophobicity, transparency and adhesiveness, thereby making this compound a good coating material for various substrates. Reports have shown that PMEA is an effective coating agent for artificial heart–lung systems [20,21]. Poly(2-methacryloyloxyethylphosphorylcholine) (PMPC) has pendant phosphorylcholine groups and a structure that is identical to that of cell membrane phospholipids, thereby making it a useful biocompatible material for suppressing protein adsorption [22]. Copolymers containing 2-methacryloyloxyethyl phosphorylcholine (MPC) units with different structures have also been utilized for long-term biomedical applications [23,24,25,26,27,28,29,30,31,32]. Inspired by these results, we theorized that an MEA/MPC copolymer would exhibit excellent biocompatibility and would be useful for numerous biomedical applications.

**Figure 1:**
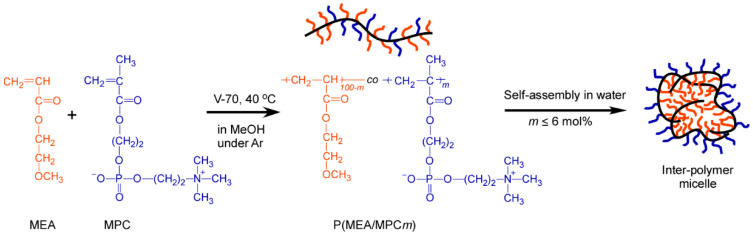
Synthesis and conceptual illustration of the self-assembly processes of the statistical copolymer, P(MEA/MPC*_m_*), in water.

In this research, biocompatible amphiphilic copolymers like P(MEA/MPC*_m_*), which were composed of hydrophobic 2-methoxyethyl acrylate (MEA) and hydrophilic MPC, were prepared via conventional free-radical polymerization. Here, *m* was equivalent to 6, 12 and 46 mol%, and was indicative of the number of hydrophilic MPC units present within the copolymer, as indicated via ^1^H NMR. In addition, the self-association behavior of P(MEA/MPC*_m_*) was investigated in water. In this case, P(MEA/MPC_12_) and P(MEA/MPC_46_) were dissolved in water as unimers, whereas P(MEA/MPC_6_) formed interpolymer micelles containing a PMEA hydrophobic domain and a PMPC shell (Figure 1).

## 2. Materials and Methods

### 2.1. Materials

2-Methoxyethyl acrylate (MEA, >98%), which had been obtained from Wako Pure Chemical (Osaka, Japan), was removed with a Sigma-Aldrich (St. Louis, MI, USA) inhibitor remover column. 2-Methacryloyloxyethyl phosphorylcholine (MPC), which had been obtained from NOF Corp. (Tokyo, Japan), was purified using a previously reported method [33]. 2′-Azobis(4-methoxy-2,4-dimethylvaleronitrile) (V-70, >95%), which had been purchased from Wako Pure Chemical (Osaka, Japan), was used as received without any further purification. The pyrene (97%) purchased from Wako Pure Chemical was purified via recrystallization using methanol. Methanol was dried using 4Å molecular sieves and purified via distillation. Water was purified with an ion-exchange column system.

### 2.2. Monomer Reactivity Ratio and Polymerization Kinetics

The monomer reactivity ratio was determined using the Fineman–Ross method [34]. Here, MEA, MPC and V-70 were dissolved in methanol with feed ratios of MPC ranging from 10% to 90% (([MEA] + [MPC])/[V-70] = 100/0.4). A small amount of methanol-*d*_4_ was added to the solution, which was transferred to NMR tubes and purged with argon gas for 30 min. Polymerization was performed in an oil bath at 40 °C. Quenching procedures were conducted via rapid cooling in an ice bath when the monomer conversion, which was monitored via ^1^H NMR, was less than 20%. The reaction mixture was dialyzed (MWCO:500 ~ 1000) using pure water for one night. Next, the solvent was removed via evaporation, and then the residue obtained was subsequently dissolved in 300 µL of methanol-*d*_4_ before ^1^H NMR analysis was conducted. The MEA and MPC contents in the copolymer were determined by comparing the ^1^H NMR integral intensities of the peaks attributed to the pendant methylene protons in MEA (3.62 ppm) and MPC (3.72 ppm) (Figure S2 and Table S1).

The ratio (*m*_MEA_/*m*_MPC_ = *f*) of the MEA and MPC contents in the copolymer was determined using the following equation:

(1) $$\frac{m_{\mathrm{MEA}}}{m_{\mathrm{MPC}}}=\frac{{\left[M_{\mathrm{MEA}}\right]}_{0}}{{\left[M_{\mathrm{MPC}}\right]}_{0}}\times \frac{r_{\mathrm{MEA}}{\left[M_{\mathrm{MEA}}\right]}_{0}+{\left[M_{\mathrm{MPC}}\right]}_{0}}{r_{\mathrm{MPC}}{\left[M_{\mathrm{MPC}}\right]}_{0}+{\left[M_{\mathrm{MEA}}\right]}_{0}}$$

where *m*_MEA_ and *m*_MPC_ are the molar contents of MEA and MPC in the copolymer, [M_MEA_]_0_ and [M_MPC_]_0_ are the initial molar concentration of the MEA and MPC monomers before polymerization, and *r*_MEA_ and *r*_MPC_ are the monomer reactivity ratios of MEA and MPC. Equation (1) can be rewritten as:

(2) $$\frac{F\left(f-1\right)}{f}=\frac{r_{\mathrm{MEA}}F^{2}}{f}-r_{\mathrm{MPC}}$$

where *F* is the ratio of the initial molar concentrations of MEA and MPC (=[M_MEA_]_0_/[M_MPC_]_0_). A plot of *F*(*f* − 1)/*f* as the ordinate and (*F*^2^/*f*) as the abscissa results in a straight line whose slope is represented by *r*_MEA_, and the intercept is the negative *r*_MPC_ value.

The relationship between the extent of the monomer conversion and the polymerization time was also studied. Here, MEA (65.5 mg, 0.503 mmol), MPC (148.5 mg, 0.503 mmol) and V-70 (1.28 mg, 0.00412 mmol) were dissolved in 0.5 mL of methanol-*d*_4_ ([MEA]/[MPC]/[V-70] = 50/50/0.4) before the solution was transferred to an NMR tube and purged with argon gas for 30 min. The polymerization reaction was performed at 40 °C, and NMR analysis was used to monitor the process. The resulting NMR spectra were recorded at different polymerization times to determine the extent of conversion of the MEA and MPC monomers by comparing the integral intensity ratios of the vinyl protons observed at 6.37 and 5.62 ppm, respectively, before and after polymerization.

### 2.3. Preparation of the MEA Homopolymer

PMEA was synthesized via the conventional free-radical polymerization reaction using the following procedure. First, MEA (0.260 g, 2.0 mmol) and V-70 (2.45 mg, 0.008 mmol) were dissolved in methanol (2.0 mL), and the mixture was purged with Ar gas in 30 min before polymerization was conducted at 40 °C for 18 h. We noted that MEA conversion was 66.7%. After polymerization, the solution was dialyzed using methanol for one day and pure water for an additional 24 h. Mild precipitation was observed after dialysis. The solution was transferred to a 20-mL glass bottle (Figure S3a) and was subjected to dynamic light scattering (DLS) as a means of monitoring the formation of PMEA polymer micelles. After a portion of the aqueous solution was evaporated under vacuum, the residue obtained was dissolved in methanol-*d*_4_ or the mobile phase to prepare samples for ^1^H NMR analysis or size exclusion chromatography (SEC), respectively.

### 2.4. Preparation of the P(MEA/MPC_m_) Copolymer

Statistical copolymers (i.e., P(MEA/MPC*_m_*)) were synthesized via conventional free-radical polymerization in methanol using three MPC feed mol% values, namely, 5, 10 and 40 mol% (Figure 1). A typical procedure for the synthesis of P(MEA/MPC*_m_*), with *m* in the feed of 10 mol%, was as follows: first, MEA (0.240 g, 1.84 mmol), MPC (0.0594 g, 0.201 mmol) and V-70 (25.2 mg, 0.082 mmol) were dissolved in methanol (2.0 mL). The mixture was then purged with Ar gas for 30 min and subsequently stirred at 40 °C for 18 h. After polymerization, the reaction mixture was dialyzed using methanol for one day and then pure water for an additional 24 h. After dialysis, the P(MEA/MPC_6_) solution obtained was cloudy, indicating the formation of polymer micelles. Clear liquids were obtained in other cases, i.e., for P(MEA/MPC_12_) and P(MEA/MPC_46_) (Figure S3). The polymer solutions after dialysis were used for experiments focused on determining the association behavior of the copolymers in water. ^1^H NMR spectroscopy was conducted after purification to estimate the MEA and MPC contents of the copolymers obtained. A portion of the solution was freeze-dried, and the residue was dissolved in methanol or methanol-*d*_4_ to prepare a solution for further experimentation.

### 2.5. Measurements

^1^H NMR spectroscopy was performed on a JNM-ECZ 400 MHz spectrometer (JEOL, Tokyo, Japan) using D_2_O or methanol-*d*_4_ as the deuterated solvents. Spin–spin relaxation times (*T*_2_) were measured using the Car–Purcell–Meiboom–Gill method. Echoes were observed at the 180° pulse, and the amplitude of the successive echoes decayed exponentially with a time constant equal to *T*_2_ [35]. Array parameters of the delay list, which included 16 points, were set for conducting the measurements. The data were analyzed via the Weight Linear Spin Lock method using Delta v5.3.1 software (JEOL, Tokyo, Japan). SEC was performed using an instrument equipped with a 7.0-μm bead GF-7M HQ column from Shodex (Tokyo, Japan) and a Shodex RI-101 refractive index (RI) detector operating at 40 °C. The elution phase was methanol containing 0.1 M lithium perchlorate at a flow rate of 0.6 mL/min. Poly(ethylene oxide) was used to prepare the universal standard curve to determine the number-average molecular weight (*M*_n_) and the molecular weight distribution (*Đ*). Sample solutions were filtered using a 0.2-μm pore size membrane conducting the relevant measurements. The hydrodynamic radius (*R*_h_) and light scattering intensity (LSI) of P(MEA/MPC*_m_*) in water were obtained using a Malvern (Worcestershire, UK) Zetasizer 7.11 equipped with a 4-mW He–Ne laser at 25 °C. The wavelength of the light source was 632.8 nm. The data obtained were analyzed using a Malvern (Worcestershire, UK) Zetasizer 7.11. The polymer concentration (*C*_p_) of P(MEA/MPC_m_) in aqueous solution was fixed at 1.0 g/L. The sample solutions were filtered with a 0.45-μm pore size membrane before conducting the analysis. Transmission electron microscopy (TEM) was performed using a JEOL JEM-2100F (Tokyo, Japan) with an acceleration voltage of 160 kV. The samples were prepared by adding the respective aqueous polymer solution dropwise onto a JEOL (Tokyo, Japan) 150-mesh copper TEM grid, and subsequently staining it with 0.1 wt.% phosphotungstic acid aqueous solution. Next, the samples were dried under vacuum conditions at room temperature. Static light scattering (SLS) measurements were conducted using a DLS-7000 Otsuka Electronics Photal^TM^ (Osaka, Japan) at 25 °C, with a He–Ne laser (10.0 mW at 632.8 nm) as the light source. The weight-average molecular weight (*M*_w_), the radius of gyration (*R*_g_) and the second virial coefficient (*A*_2_) of the P(MEA/MPC_6_) polymer micelles in water were estimated from Zimm plots constructed using data derived from aqueous polymer solutions at two different concentrations (i.e., 0.25 and 0.5 g/L). The *M*_w_ and *R*_g_ values of all copolymers in methanol in the random-coil state were calculated from the Zimm plot at 10 g/L. The RI increment (d*n*/d*C*_p_) values were determined using a DRM-3000 differential refractometer (Otsuka Electronics Co., Osaka, Japan) at 25 °C. The d*n*/d*C*_p_ values were 0.0619, 0.0768 and 0.104 mL/g for P(MEA/MPC*_m_*) in methanol, with *m* values of 5, 10 and 40 mol%, respectively, in the feed. These values were used for conducting SLS analysis of the corresponding samples. The CAC of the polymer aqueous solution was determined using pyrene as the fluorescent probe. The fluorescence spectra of the pyrene/polymer aqueous solutions were recorded with an F-2500 fluorescence spectrophotometer from Hitachi (Tokyo, Japan).

## 3. Results and Discussion

### 3.1. Determination of the Monomer Reactivity Ratio

**Figure 2:**
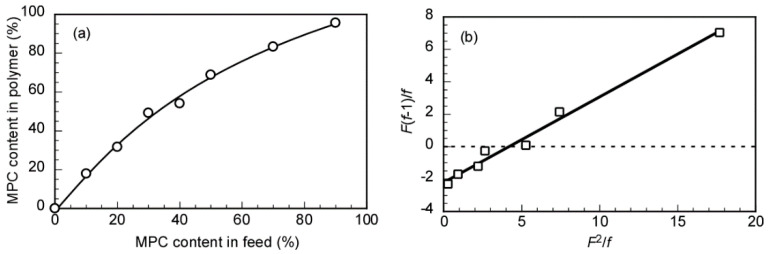
(**a**) Relationship between the 2-methacryloyloxyethyl phosphorylcholine (MPC) content in the copolymer and the feed. (**b**) The graphic representation of the Fineman–Ross equation, with *F*(*f*-1)/*f* as a function of (*F*^2^/*f*).

Conventional free-radical polymerizations of equimolar concentrations of MEA and MPC were conducted in the presence of the V-70 initiator at 40 °C. Here, the monomer concentration was estimated based on the observed decrease in the integral intensity of the vinyl protons in the ^1^H NMR spectra, at 6.37 and 5.62 ppm for MEA and MPC, respectively. The time conversion plots (Figure S1a) indicated that MPC conversion rapidly reached 90% after 120 min and continued to increase to 99% after 300 min. Conversely, the MEA reaction was notably slower, as MEA conversion after 300 min was 82%. These results were consistent with the reported monomer reactivity findings obtained using the Fineman–Ross method (Figure 2b). The first-order kinetic plots showed that both plots were linearly related to the polymerization time during the early stages of the polymerization reaction until the 100-min time mark, indicating that the propagating radical concentration was constant (Figure S1b). The late stages of the polymerization reaction (i.e., after 100 min) produced plots with a downward curvature, suggesting that the propagating radical concentration had decreased. Thus, a straight fitted line was obtained from the Fineman–Ross plots, in which the slope and intercept represented the monomer reactivity ratios of MEA and MPC, respectively. The monomer reactivity ratios of MEA and MPC were 0.53 and 2.21, respectively. Since the reactivity of MPC was almost four times higher than that of MEA, the probability of MPC being incorporated into the copolymer was much higher. Figure 2a also showed that the MPC mol% in the copolymer was always higher than the percentage of MPC in the feed ratio. The reactivity ratios of MEA and MPC were in good agreement with the values observed during the copolymerization of methyl acrylate (MA) and MMA, both of which possessed similar structures. Grassie et al. [36] reported that the reactivity ratios of MA and MMA were 0.35 and 1.8, respectively, during copolymerization at 65 °C. For MA and MMA, these values were 0.47 and 2.3, respectively, during copolymerization at 130 °C, indicating that the reactivity ratio of MMA was almost four to five times higher than MA, and was temperature-independent. From the results, *r*_MEA_ × *r*_MPC_ was 1.17, which demonstrated that an almost random monomer sequence was the main feature of the copolymerization process in these compounds. Note, however, that the repeating units in the copolymer were composed of more MPC, since MPC was significantly more active than MEA.

### 3.2. Preparation of PMEA and P(MEA/MPC_m_)

**Figure 3:**
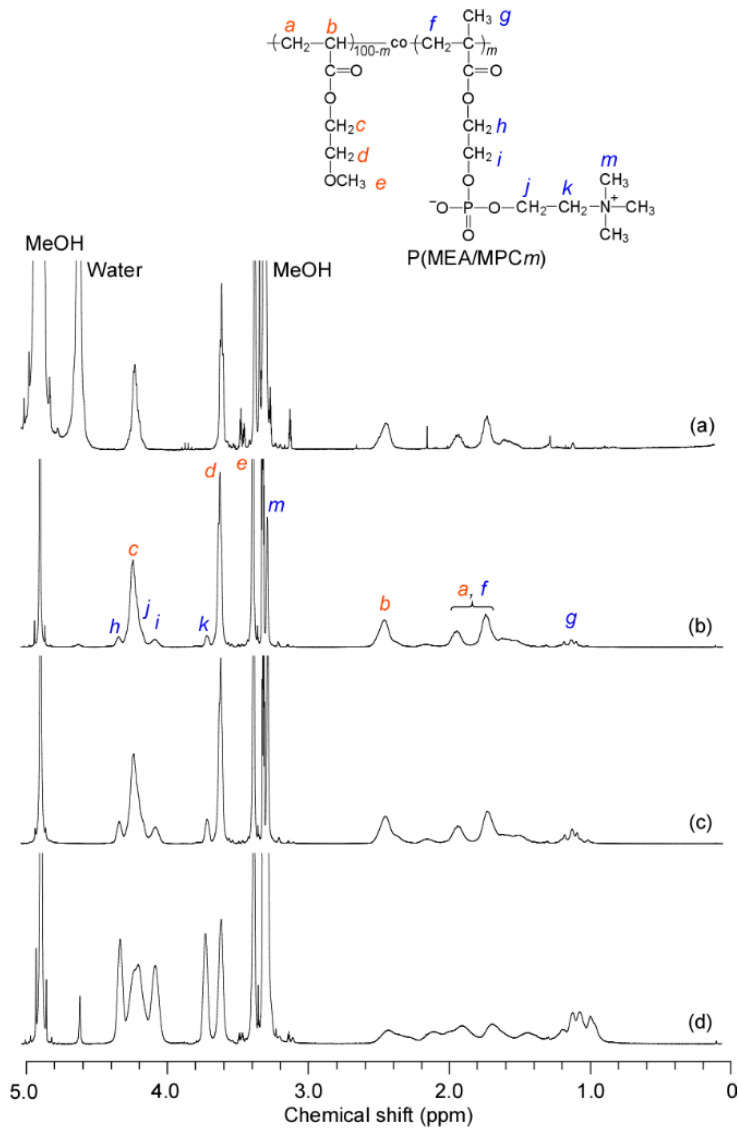
^1^H NMR spectra for (**a**) PMEA, (**b**) P(MEA/MPC_6_), (**c**) P(MEA/MPC_12_), and (**d**) P(MEA/MPC_46_) in methanol-*d*_4_ at room temperature.

PMEA was prepared via conventional free-radical polymerization reactions. The conversion of MEA, which was estimated from the corresponding NMR data, was 66.7%. The *M*_n_ and *Đ* values were 1.32 × 10^4^ g/mol and 2.54, respectively. The P(MEA/MPC*_m_*) copolymers were prepared with three different compositions (i.e., *m* = 6, 12 and 46 mol%) via conventional free-radical polymerization reactions. The conversions of the MEA and MPC monomers were in the ranges of 72–89% and 98–100%, respectively. The MPC content in the copolymer was estimated from ^1^H NMR spectra obtained in methanol-*d*_4_, using the integral intensity ratio of the peaks attributed to the pendant methylene protons in the MEA and MPC units; these peaks were observed at 3.62 and 3.72 ppm for MEA and MPC, respectively (Figure 3 and Table S2). SEC measurements were conducted for all polymers, and unimodal curves were observed for all samples with *Đ* values between 2.0 and 2.6 (Figure S4). We theorized that this was due to the occurrence of uncontrolled polymerization. The SEC charts indicated that P(MEA/MPC_46_) had the longest retention time (Figure S4), which was possibly due to unexpected interactions between the SEC column and the copolymer. The *M*_w_ values of a single polymer chain were determined using SLS measurements in methanol. Unimodal distribution was observed in the DLS results for the methanolic solutions of the copolymer, with small *R*_h_ values between 9 and 10 nm (Figure S5). The *R*_g_ and *M*_w_ values of the copolymers were estimated via conducting SLS measurements in methanol (Figure S6). Here, we noted that the *R*_g_/*R*_h_ values for all the copolymers were less than or equal to 1.6, indicating that the polymers were dissolved in methanol as with a large polydispersity index (PDI) [37]. In particular, the *R*_g_/*R*_h_ value for P(MEA/MPC_46_) was associated with a large degree of error due to the bimodal *R*_h_ distribution (Figure S5). These features made it possible to determine the molecular weight of the individual polymer chains using SLS measurements in methanol. The characteristics of all samples are summarized in Table 1.

### 3.3. Self-Association Behavior of P(MEA/MPC_m_) in Water

**Figure 4:**
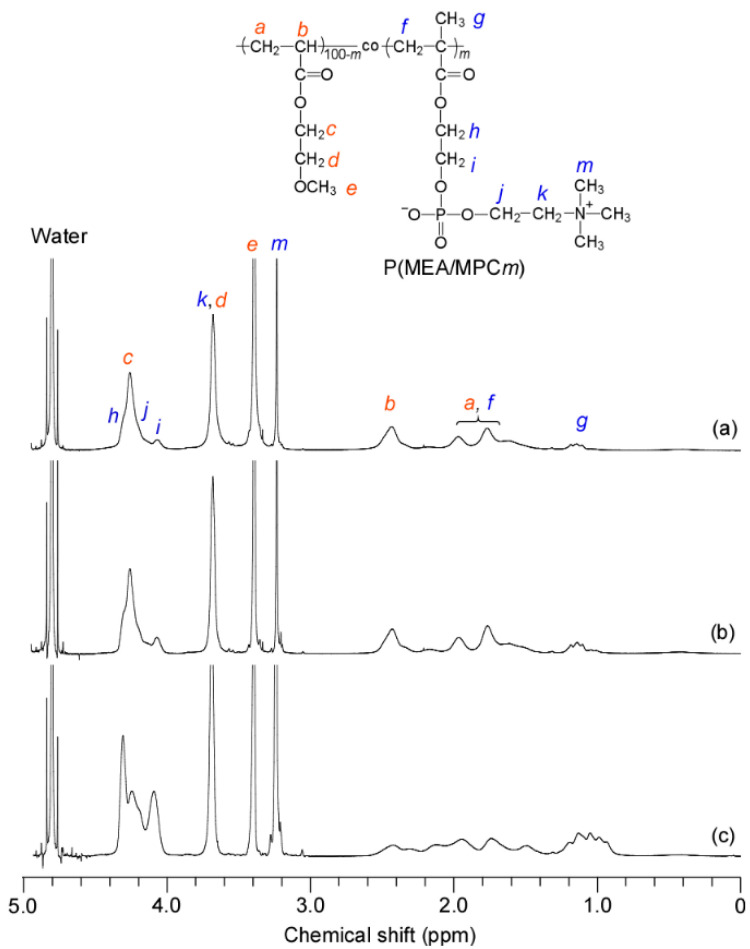
^1^H NMR spectra for (**a**) P(MEA/MPC_6_), (**b**) P(MEA/MPC_12_), and (**c**) P(MEA/MPC_46_) in D_2_O at room temperature.

NMR measurements were recorded for all copolymers in D_2_O (Figure 4). Here, the pendant methylene proton signals of the MEA units were observed at 3.68 ppm in D_2_O, which was shifted downfield from their position at 3.62 ppm in methanol-*d*_4_. As a result, the signals of the MEA and MPC units overlapped at 3.72 ppm.

**Figure 5:**
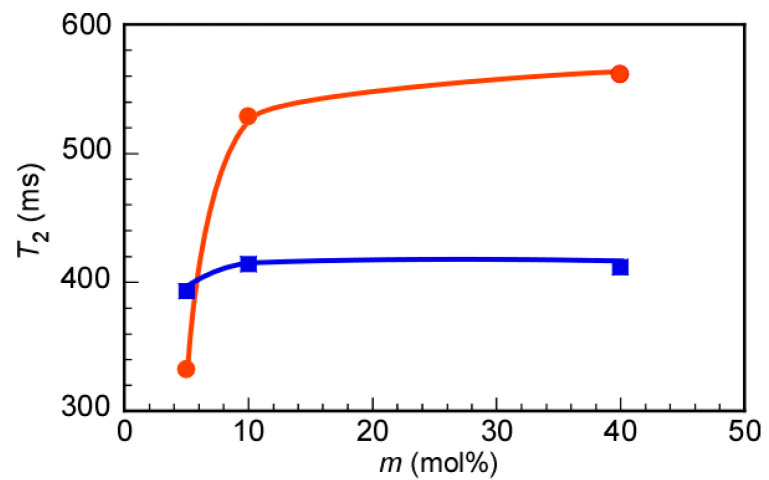
Spin–spin relaxation time (*T*_2_) for the pendant methyl protons in the MEA unit at 3.39 ppm (●) and the MPC unit at 3.23 ppm (■) as a function of the MPC content (*m*) in P(MEA/MPC*_m_*) in D_2_O at 25 °C.

The dynamic motion of individual molecular segments could be estimated by measuring the ^1^H NMR spin–spin relaxation time (*T*_2_) [38,39]. We noted that when the polymer micelles were formed, the motion of the protons relative to the hydrophobic MEA unit was restricted to the hydrophobic domain of the polymer micelle. As a result, restrictions in the motion of the MEA units decreased the *T*_2_ values. Here, the *T*_2_ values were estimated for the methyl protons at 3.39 and 3.23 ppm in the MEA and MPC units of the copolymers, respectively, in D_2_O (Figure 5). We noted that the *T*_2_ values of the pendant methyl protons in the MEA units decreased with decreasing *m*, as exemplified by the smallest value of 332 ms, which was obtained when *m* was 6 mol%. For P(MEA/MPC_12_) and P(MEA/MPC_46_), the *T*_2_ values (namely, 528 and 561 ms, respectively) were greater than those observed for P(MEA/MPC_6_). The *T*_2_ values did not significantly change when *m* increased from 12 to 46 mol%. These observations indicated that the motion of the hydrophobic MEA units in P(MEA/MPC_6_) was restricted due to the formation of the hydrophobic domain. In contrast, the motion of the MEA units in P(MEA/MPC_12_) and P(MEA/MPC_46_) was free, since these copolymers were dissolved as unimers in water. The *T*_2_ values of the pendant methyl protons in the MPC units were almost the same, regardless of the *m* value; for P(MEA/MPC_6_), P(MEA/MPC_12_), and P(MEA/MPC_46_), these values were 393, 414, and 412 ms, respectively. As noted earlier, the hydrophilic MPC units were arranged on the surface of the micelles when P(MEA/MPC_6_) formed polymer micelles, whereas P(MEA/MPC_12_) and P(MEA/MPC_46_) were dissolved as unimers. Since the MPC units were always exposed to water, their motion remained almost the same for all samples. The *T*_2_ values of the pendant methyl protons in the MPC units of P(MEA/MPC_6_) decreased slightly as some of the MPC units may have been incorporated into the hydrophobic domain due to the statistical sequence in the copolymer’s structure. These results provided additional evidence of the formation of polymer micelles of P(MEA/MPC_6_) in water.

**Figure 6:**
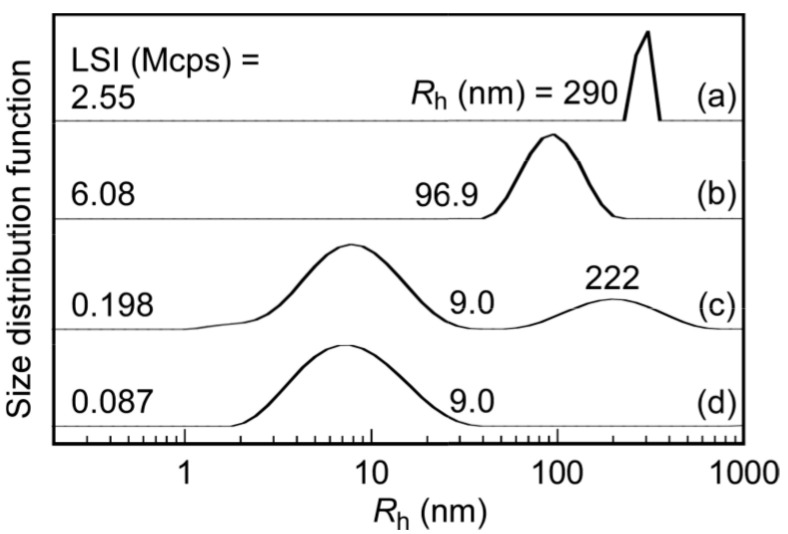
Hydrodynamic radius (*R*_h_) distributions for (**a**) PMEA, (**b**) P(MEA/MPC_6_), (**c**) P(MEA/MPC_12_), and (**d**) P(MEA/MPC_46_) in pure water at *C*_p_ = 1.0 g/L at 25 °C.

The self-association behavior of P(MEA/MPC*_m_*) in water was studied by conducting DLS measurements on the copolymer aqueous solutions at *C*_p_ = 1.0 g/L (Figure 6). Here, the *R*_h_ distribution values for PMEA were established as a reference sample. Small amounts of precipitate were obtained after the purification of PMEA via dialysis, which contained high molecular weight chains (i.e., large aggregates) that were removed via filtration. The PMEA chains with lower molecular weight values did not precipitate, as they were dispersed in water during dialysis. For the aqueous solution of P(MEA/MPC_46_), unimodal distribution was observed with a small *R*_h_ value of 9.0 nm, suggesting that P(MEA/MPC_46_) was dissolved in water as the unimer. Interestingly, bimodal distribution was observed in P(MEA/MPC_12_), which contained one peak with the same *R*_h_ value (i.e., 9.0 nm) as P(MEA/MPC_46_) in water and another peak with a higher *R*_h_ (i.e., 222 nm) value. In this case, the formation of interpolymer associates composed of some of the hydrophobic MEA units in the copolymer was identified as the reason for these observations.

Generally, weak hydrophobic interactions were noted when the hydrophobic content in the polymer was insufficient, resulting in the formation of only a few micelles. For PMEA and P(MEA/MPC_6_), unimodal distribution was noted with *R*_h_ values of 290 and 96.9 nm, respectively, indicating that P(MEA/MPC_6_), with its 94 mol% of the hydrophobic MEA unit, easily formed interpolymer aggregates in water. The radius of the polymer aggregates increased when PMEA was employed, resulting in self-association to generate larger aggregates in water. All of the above mentioned findings were supported by the results obtained via LSI analysis of these samples. Here, the precipitation observed after dialysis using pure water was evidence that PMEA could form very large aggregates. Only the supernatant was used for DLS analysis, and the estimated concentration of the PMEA aqueous solution was believed to be lower than the true value. This was proposed as the possible reason for the lower LSI values observed for PMEA relative to P(MEA/MPC_6_) in water at the same concentration. By extension, the LSI values of both PMEA and P(MEA/MPC_6_) were greater than those obtained for P(MEA/MPC_12_) and P(MEA/MPC_46_) due to the formation of polymer micelles. Since P(MEA/MPC_46_) was dissolved as random coils in water, the smallest recorded LSI value in our study was 0.087 Mcps. As noted, the aqueous solution of PMEA became cloudier after a few days, whereas the P(MEA/MPC_6_) aggregates exhibited increased stability over the same observation time. The stability of the P(MEA/MPC_6_) aggregates during dilution was confirmed via DLS measurements. Here, the *R*_h_ values of P(MEA/MPC_6_) were nearly constant and independent of *C*_p_ in the 0.05–5 g/L region (Figure S7). Note, however, that lower concentrations were difficult to measure due to the low LSI values associated.

**Figure 7:**
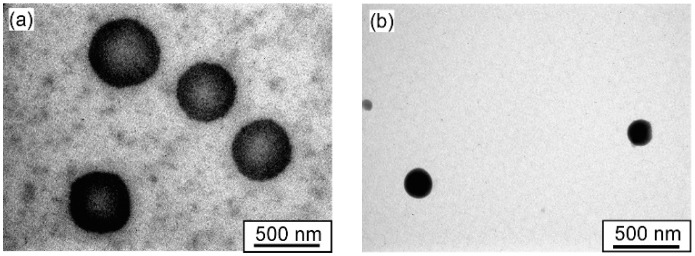
Transmission electron microscopy (TEM) images for (**a**) PMEA and (**b**) P(MEA/MPC_6_) at *C*_p_ = 1.0 g/L in water.

On another note, spherical objects were observed for PMEA and P(MEA/MPC_6_) copolymers using TEM (Figure 7), with average radii of 240 and 103 nm for PMEA and P(MEA/MPC_6_), respectively. Additional TEM observations for P(MEA/MPC_6_) are shown in Figure S8.

**Figure 8:**
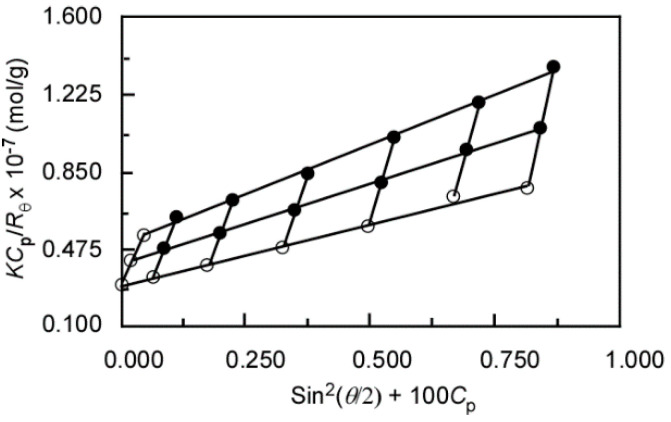
Zimm plots of P(MEA/MPC_6_) in water at 25 °C.

The characterization of the P(MEA/MPC_6_) micelles in water was conducted via SLS measurements (Figure 8). Here, the d*_n_*/d*_C_*_p_ value for P(MEA/MPC_6_) in water was 0.0651 mL/g, and was subsequently used during SLS analysis to estimate the apparent *M*_w_ of the P(MEA/MPC_6_) micelles. The aggregation number (*N*_agg_), which is the number of individual polymer chains present in one micelle, was calculated from the *M*_w_ values of the polymer micelle in water and the individual polymer chain in methanol obtained from SLS measurements. The *N*_agg_ of P(MEA/MPC_6_) micelle was 143 in water, indicating the formation of interpolymer micelles. It was speculated that the hydrophobic interactions between the MEA units in the individual polymer chains were too weak to facilitate the self-folding of a single chain. For P(MEA/MPC_6_) with 94 mol% of the relatively hydrophobic MEA units in the copolymer’s composition, hydrophobic interactions between multiple chains resulted in interpolymer aggregates. This phenomenon was also seen in the aqueous solution of random copolymers of sodium 2-(acrylamido)-2-methylpropanesulfonate and *n*-dodecyl methacrylamide, and in the copolymers of poly(ethylene glycol) methacrylate and *n*-dodecyl methacrylate. Here, interpolymer aggregates were formed when the hydrophobic content of the copolymer exceeded 50 mol% [10,11].

Theoretically, the shape and size distribution of the aggregates can be estimated using *R*_g_/*R*_h_. For instance, a value of 0.778 represents a rigid sphere with a narrow distribution, whereas a value of 1.0 is associated with spherical aggregates, and values higher than 2 are often seen in rodlike structures [37,40]. In our study, the *R*_g_/*R*_h_ value for P(MEA/MPC_6_) in water was 0.95, indicating that the polymer micelles were spherical (Table 2). The *A*_2_ value provides information about the affinity of solute molecules with the solvent of interest [41]. For our case, the *A*_2_ value obtained from the SLS analysis of P(MEA/MPC_6_) in water was 2.5 × 10^−5^ cm^3^ g^−2^ mol. This positive value was proof that the hydrophilic MPC unit had completely covered P(MEA/MPC_6_), thereby facilitating its solubility in water. The *A*_2_ value for the MPC homopolymer was reported as 2.5 × 10^−^^4^ cm^3^ g^−2^ mol in aqueous solution [42]. The *A*_2_ value for P(MEA/MPC_6_) was 10 times lower than that of the MPC homopolymer, which was attributed to the composition of the copolymer; here, the copolymer consisted of 94 mol% of the hydrophobic MEA unit.

**Figure 9:**
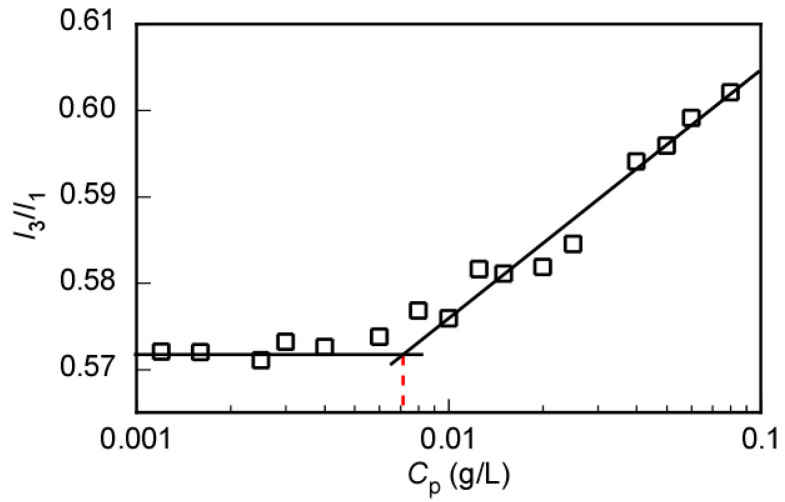
Fluorescence intensity ratio (*I*_3_/*I*_1_) of pyrene in the presence of P(MEA/MPC_6_) plotted against the polymer concentration (*C*_p_). *I*_3_ and *I*_1_ are the fluorescence intensities of the third and the first vibronic peaks, respectively, in the pyrene emission spectra recorded at the excitation wavelength of 334 nm.

The CAC value of P(MEA/MPC_6_) was determined via fluorescence spectroscopy using pyrene as the probe molecule. Pyrene is hydrophobic and its fluorescence spectrum is heavily dependent on the polarity of the surrounding environment. The intensity ratio (*I*_3_/*I*_1_) between the third and the first vibronic peaks in the pyrene emission spectra can be used as a measure of the environmental polarity [43]. Therefore, the fluorescence spectra of pyrene in the presence of P(MEA/MPC_6_) at varying *C*_p_ were recorded at the excitation wavelength of 334 nm (Figure S9). The changes in the *I*_3_/*I*_1_ ratio in the pyrene emission spectra were plotted as a function of *C*_p_ to determine CAC (Figure 9). We noted that the copolymers did not form micelles below the CAC. The solubility of pyrene in water ensured that the resulting fluorescence spectrum remained almost unchanged (*I*_3_/*I*_1_ ≈ 0.572). This calculated value was nearly the same as that obtained for pyrene in the absence of the copolymer (*I*_3_/*I*_1_ ≈ 0.562), demonstrating that there was no discernible difference in the polarity of the environment surrounding pyrene as the P(MEA/MPC_6_) copolymer was locked in random coils at low concentrations in water. However, the formation of copolymer micelles above the CAC meant that the pyrene molecules were entrapped in the hydrophobic domains. As such, the pyrene molecules were surrounded by a non-polar environment. The number of pyrene molecules in the hydrophobic domain increased when *C*_p_ rose. Consequently, the *I*_3_/*I*_1_ value gradually increased to a maximum of 0.602 at *C*_p_ = 0.08 g/L. The CAC value of P(MEA/MPC_6_) in water was determined at the inflection point as 0.0082 g/L. Unlike the formation of unimer micelles independent of *C*_p_, P(MEA/MPC_6_) was incapable of aggregation in dilute aqueous solutions. Interpolymer association occurred when the P(MEA/MPC_6_) concentration increased to a certain level. Therefore, the CAC value was determined as the *C*_p_ at which the interpolymer micelles started to form.

## 4. Conclusions

Biocompatible, amphiphilic, statistical copolymers, P(MEA/MPC*_m_*), which were composed of hydrophobic MEA and hydrophilic MPC units, were prepared via conventional free-radical polymerization reactions. P(MEA/MPC_12_) and P(MEA/MPC_46_) possessed a high MPC content, were dissolved as unimers, and did not aggregate in water. On the other hand, P(MEA/MPC_6_) formed uniform micelles composed of a PMEA core and a PMPC shell, with *R*_h_ and *N*_agg_ values of 96.9 nm and 133, respectively, due to the hydrophobic interactions of the MEA units. The mobility of the MEA units in P(MEA/MPC_6_) was restricted, as confirmed via the observed ^1^H NMR spin–spin relaxation times. A slight decrease in the motion of the MPC units was also observed and was attributed to the entrapment of some MPC units in the hydrophobic domain; the associated interpolymer aggregates were covered by phosphorylcholine groups. From these results, we concluded that P(MEA/MPC*_m_*) copolymers with *m* ≤ 6 mol% formed micelles, and, as such, were well suited for biomedical applications.

## Figures and Tables

**Table 1:** Characteristics of the P(MEA/MPC*_m_*) copolymers.

*m ^a^*	*M*_n_(SEC) *^b^* × 10^4^ (g/mol)	*Đ ^b^*	*M*_w_(SLS) *^c^* × 10^5^ (g/mol)	*R*_h_*^d^* (nm)	*R*_g_*^c^* (nm)	*R*_g_/*R*_h_
6	1.41	2.61	2.36	9.6	15.5	1.6
12	1.46	2.24	1.53	10.0	14.8	1.5
46	2.69	2.04	0.73	9.2	12.8	1.4

*^a^ m* indicates the MPC content (mol%) in the copolymer, as estimated using NMR data obtained in methanol-*d*_4_. *^b^* The number-average molecular weight (*M*_n_) and molecular weight distribution (*Đ*) were estimated via SEC analysis. *^c^* The apparent weight-average molecular weight (*M*_w_) and the radius of gyration (*R*_g_) were estimated from SLS measurements in methanol. *^d^* The hydrodynamic radius (*R*_h_) was obtained from DLS measurements in the methanolic solutions of the copolymers.

**Table 2:** The association behavior of P(MEA/MPC_6_) in water.

Sample	*M*_w_(SLS) ^a^ × 10^−7^ (g/mol)	*R*_h_^b^ (nm)	*R*_g_^a^ (nm)	*R*_g_/*R*_h_	*R*_TEM_^c^ (nm)	*N*_agg_^d^	*A*_2_^a^ × 10^5^ (cm^3^ g^−2^ mol)	CAC ^e^ (g/L)
P(MEA/MPC_6_)	3.37	96.9	92.1	0.95	103	143	2.5	0.0082

^a^ Estimated from static light scattering (SLS) analysis of the aqueous solutions. ^b^ Obtained from DLS analysis of the aqueous solutions. ^c^ Estimated via TEM. ^d^ Aggregation number (*N*_agg_) calculated from the *M*_w_ values of the polymer micelle in water and the individual polymer chains in methanol obtained from SLS measurements. ^e^ Critical aggregation concentration (CAC) estimated via the pyrene fluorescence method.

## Supplementary Materials

The following are available online at https://www.mdpi.com/2073-4360/12/8/1808/s1: Figure S1. (a) Time-conversion and (b) the pseudo first-order kinetic plots for conventional free-radical polymerization of equimolar concentrations of MEA (●) and MPC (■) in methanol at 40 °C. [M]_0_ and [M] were the monomer concentrations at a polymerization times of 0 and *t* min, respectively; Figure S2. ^1^H NMR spectra of P(MEA/MPC*_m_*) with various feed mol% of the hydrophilic MPC in methanol-*d*_4_ at room temperature; Figure S3. Photographs of (a) PMEA, (b) P(MEA/MPC_6_), (c) P(MEA/MPC_12_) and (d) P(MEA/MPC_46_) solutions after dialysis using pure water; Figure S4. SEC elution curves for (a) PMEA, (b) P(MEA/MPC_6_), (c) P(MEA/MPC_12_) and (d) P(MEA/MPC_46_) using methanol containing 0.1 M lithium perchlorate as the eluent at 40 °C; Figure S5. Hydrodynamic radius (*R*_h_) distributions for (a) P(MEA/MPC_6_), (b) P(MEA/MPC_12_) and (c) P(MEA/MPC_46_) in methanol at *C*_p_ = 10 g/L at 25 °C; Figure S6. Zimm plots of (a) P(MEA/MPC_6_), (b) P(MEA/MPC_12_) and (c) P(MEA/MPC_46_) in methanol at 25 °C; Figure S7. Hydrodynamic radius (*R*_h_) of P(MEA/MPC_6_) as a function of polymer concentration (*C*_p_) in water; Figure S8. Transmission electron microscopy (TEM) images for P(MEA/MPC_6_) at *C*_p_ = 1.0 g/L in water with different magnification; Figure S9. Fluorescence spectra of pyrene excited at 334 nm in water in the presence of P(MEA/MPC_6_) at *C*_p_ = 0.08 (solid line) and 0.0012 g/L (dashed line); Table S1. The Fineman–Ross parameters of the copolymers, as determined using ^1^H NMR measurements in methanol-*d*_4_ at room temperature; and Table S2. The composition of the copolymer, as estimated using ^1^H NMR measurements in methanol-*d*_4_ at room temperature.
