# Supplementary material for: Synthesis of Amphiphilic Statistical Copolymers Bearing Methoxyethyl and Phosphorylcholine Groups and Their Self-Association Behavior in Water
Source: Polymers (Basel). 2020 Aug 12;12(8):1808. doi: 10.3390/polym12081808 (PMC7464738; doi:10.3390/polym12081808)
Supplement: Supplementary file 1 [file polymers-12-01808-s001.pdf]

Synthesis of amphiphilic statistical copolymers bearing methoxyethyl and phosphorylcholine groups and their self-association behavior in water

Thi Lien Nguyen[1], Yuuki Kawata[1], Kazuhiko Ishihara[2], and Shin-ichi Yusa[1,*]

[1] Department of Applied Chemistry, Graduate School of Engineering, University of Hyogo, 2167 Shosha, Himeji, Hyogo 671-2280, Japan; nguyenlienk56hh@gmail.com (T.L.N), yuki_chibikitty1020@yahoo.co.jp (Y.K.)

[2] Department of Materials Engineering, School of Engineering, The University of Tokyo, 7-3-1 Hongo, Bunkyo-ku, Tokyo 113-8656, Japan; ishihara@mpc.t.u-tokyo.ac.jp

* Correspondence: yusa@eng.u-hyogo.ac.jp; Tel.: +81-79-267-4954

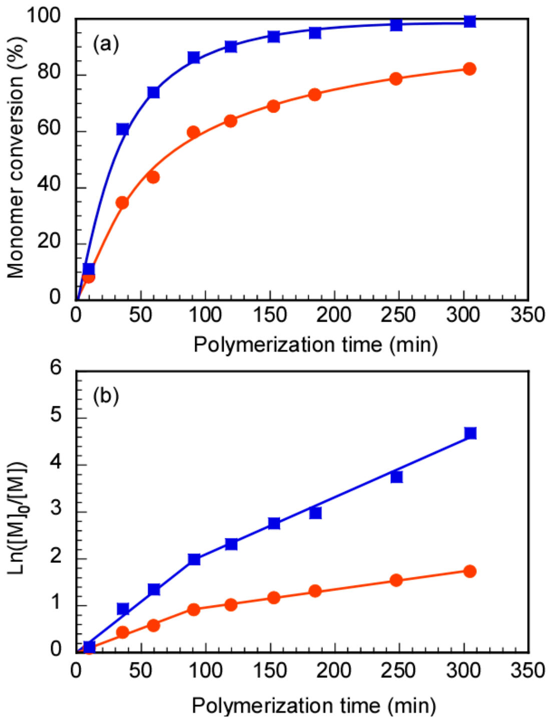

Figure S1. (a) Time-conversion and (b) the pseudo first-order kinetic plots for conventional free-radical polymerization of equimolar concentrations of MEA (●) and MPC (■) in methanol at 40°C. $[M]_0$ and [M] were monomer concentrations at polymerization times of 0 and *t* min, respectively.

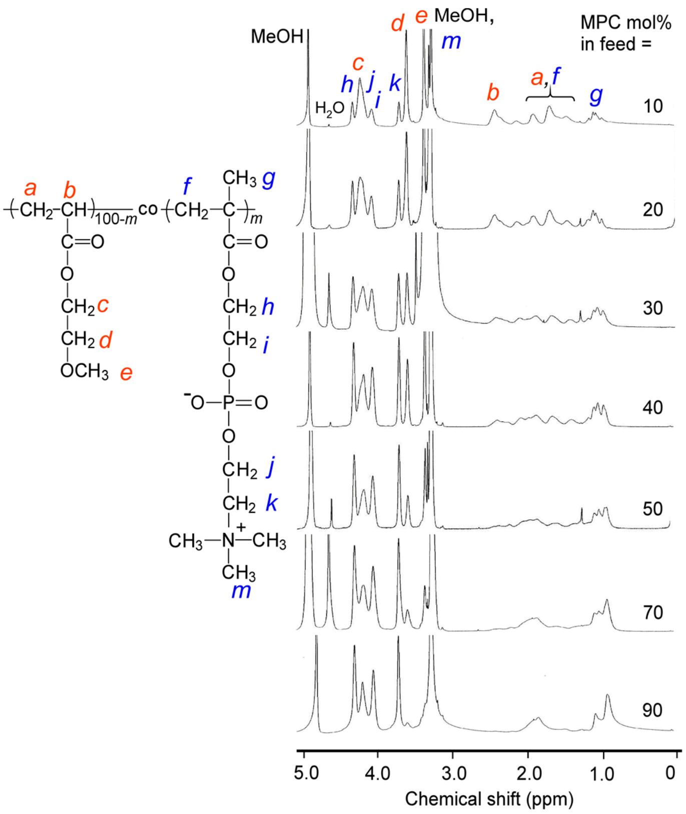

Figure S2. ^{1}H NMR spectra of P(MEA/MPC*m*) with various feed mol% of the hydrophilic MPC units in methanol-*d*4 at room temperature.

Table S1. The Fineman–Ross parameters of the copolymers, as determined via ^{1}H NMR in methanol-d_4 at room temperature

Monomer in feed (mol%)		Monomer ratio in feed	Integral intensities of monomers in the copolymers		Content ratios of monomers in the copolymer	Parameters of Fineman–Ross equation	
MEA	MPC	F = $[M_{MEA}]_0/[M_{MPC}]_0$	MEA (I_1) [a]	MPC (I_2) [b]	f = m_{MEA}/m_{MPC} = I_1/I_2	F^2/f	$F(f\text{-}1)/f$
90	10	9.00	398.7	87.1	4.58	17.69	7.03
80	20	4.00	268.8	124.8	2.15	7.43	2.14
70	30	2.33	174.2	168.8	1.03	5.28	0.07
60	40	1.50	170.5	200.5	0.85	2.65	−0.26
50	50	1.00	99.3	219.2	0.45	2.21	−1.21
30	70	0.43	43.1	215.3	0.20	0.92	−1.71
10	90	0.11	9.89	214.8	0.05	0.27	−2.30

[a] The integral intensities of the pendant methylene protons in the MEA units at 3.62 ppm. [b] The integral intensities of the pendant methylene protons in the MPC units at 3.72 ppm.

Table S2. Composition of the copolymers, as estimated via ^{1}H NMR in methanol-d_4 at room temperature

Monomer in feed (mol)		m in feed [a] (mol%)	Integral intensities in the copolymers		m in the copolymer [d] (mol%)
MEA	MPC		MEA (I_1) [b]	MPC (I_2) [c]	
1.901	0.099	4.96	9.37	0.55	5.54 ≈ 6
1.836	0.201	9.88	4.06	0.53	11.55 ≈ 12
1.201	0.800	39.97	1.75	1.49	45.99 ≈ 46

[a] m in feed = $[M_{MPC}]_0/([M_{MEA}]_0+[M_{MPC}]_0) \times 100$. [b] The integral intensities of the pendant methylene protons in the MEA units at 3.62 ppm. [c] The integral intensities of the pendant methylene protons in the MPC units at 3.72 ppm. [d] m in the copolymer = $I_2/(I_1+I_2) \times 100$.

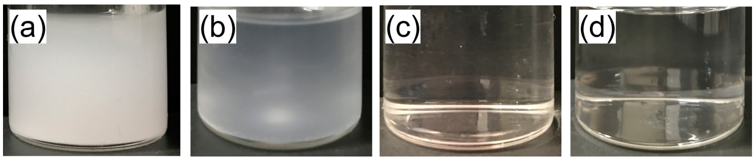

Figure S3. Photographs of (a) PMEA, (b) P(MEA/MPC_6), (c) P(MEA/MPC_{12}), and (d) P(MEA/MPC_{46}) solutions after dialysis using pure water.

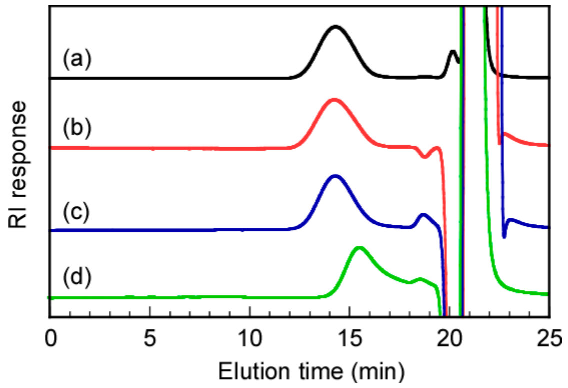

Figure S4. SEC elution curves for (a) PMEA, (b) P(MEA/MPC_6), (c) P(MEA/MPC_{12}), and (d) P(MEA/MPC_{46}) using methanol containing 0.1 M lithium perchlorate as the eluent at 40°C.

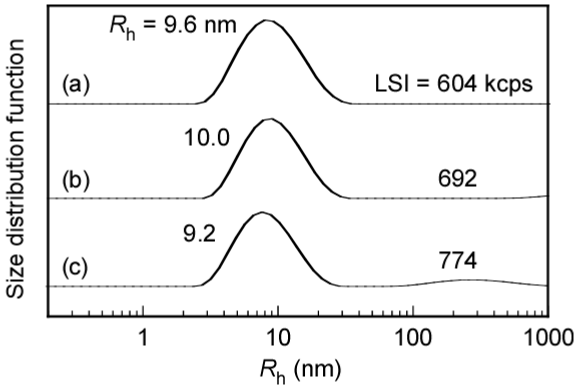

Figure S5. Hydrodynamic radius (R_h) distributions for (a) P(MEA/MPC_6), (b) P(MEA/MPC_{12}), and (c) P(MEA/MPC_{46}) in methanol at C_p = 10 g/L at 25°C.

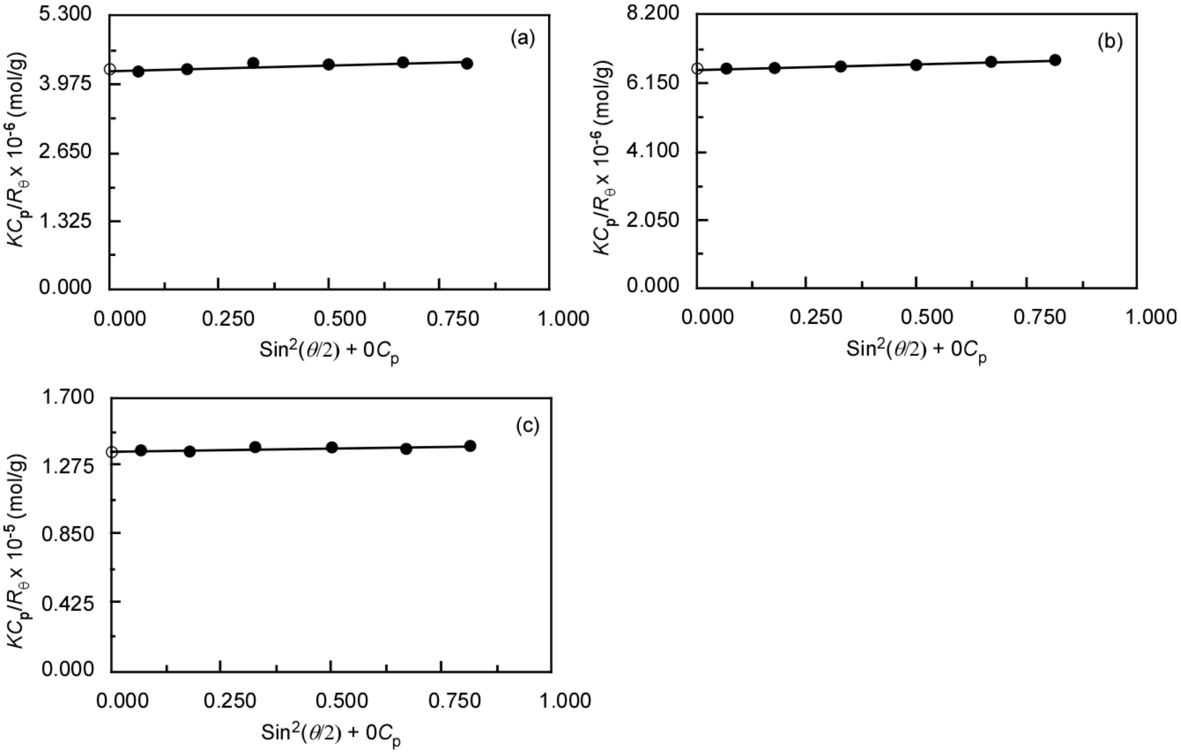

Figure S6. Zimm plots of (a) P(MEA/MPC$_6$), (b) P(MEA/MPC$_{12}$), and (c) P(MEA/MPC$_{46}$) in methanol at 25°C.

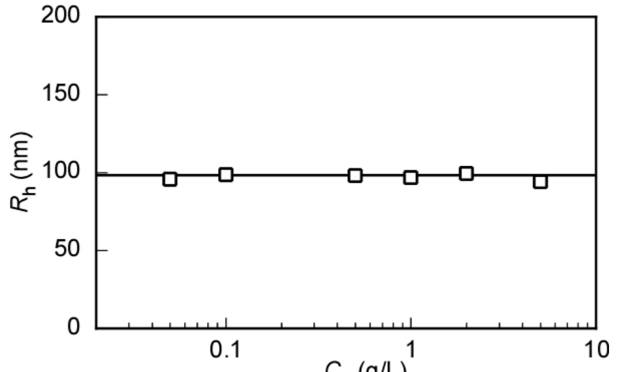

Figure S7. Hydrodynamic radius (R_h) of P(MEA/MPC$_6$) as a function of the polymer concentration (C_p) in water.

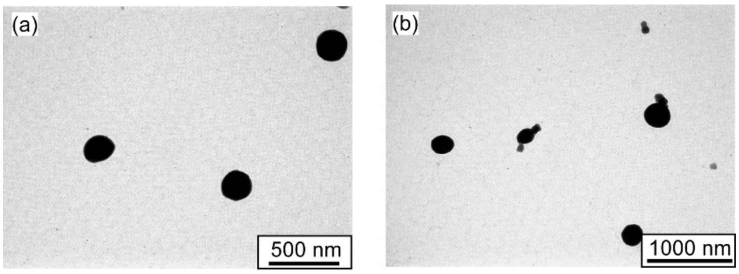

Figure S8. Transmission electron microscopy (TEM) images for P(MEA/MPC$_6$) at C_p = 1.0 g/L in water with different magnifications.

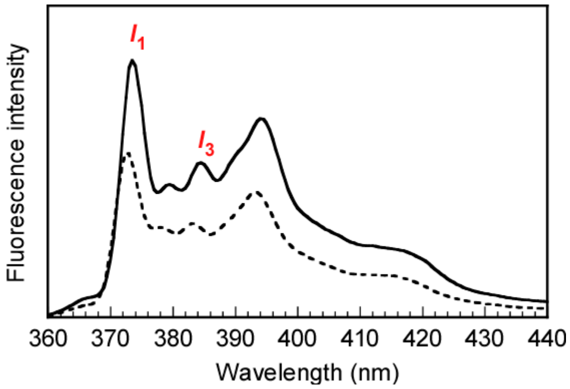

Figure S9. Fluorescence spectra of pyrene excited at 334 nm in water in the presence of P(MEA/MPC$_6$) at C_p = 0.08 (solid line) and 0.0012 g/L (dashed line).
